# Evolutionary research on the expansin protein family during the plant transition to land provides new insights into the development of Tartary buckwheat fruit

**DOI:** 10.1186/s12864-021-07562-w

**Published:** 2021-04-09

**Authors:** Wenjun Sun, Haomiao Yu, Moyang Liu, Zhaotang Ma, Hui Chen

**Affiliations:** 1 College of Life Science, Sichuan Agricultural University, Ya’an, 625014 China; 2 Joint Center for Single Cell Biology, School of Agriculture and Biology, Shanghai Jiao Tong University, Shanghai, 200240 China; 3 State Key Laboratory of Crop Gene Exploration and Utilization in Southwest China, Key Laboratory of Major Crop Diseases and Rice Research Institute, Sichuan Agricultural University, Chengdu, 611130 China

**Keywords:** Expansin, Terrestrialization, Phylogenetic, Evolutionary research, Tartary buckwheat

## Abstract

**Background:**

Plant transitions to land require robust cell walls for regulatory adaptations and to resist changing environments. Cell walls provide essential plasticity for plant cell division and defense, which are often conferred by the expansin superfamily with cell wall-loosening functions. However, the evolutionary mechanisms of expansin during plant terrestrialization are unclear.

**Results:**

Here, we identified 323 expansin proteins in 12 genomes from algae to angiosperms. Phylogenetic evolutionary, structural, motif gain and loss and Ka/Ks analyses indicated that highly conserved expansin proteins were already present in algae and expanded and purified after plant terrestrialization. We found that the expansion of the FtEXPA subfamily was caused by duplication events and that the functions of certain duplicated genes may have differentiated. More importantly, we generated space-time expression profiles and finally identified five differentially expressed *FtEXPs* in both large and small fruit Tartary buckwheat that may regulate fruit size by responding to indoleacetic acid.

**Conclusions:**

A total of 323 expansin proteins from 12 representative plants were identified in our study during terrestrialization, and the expansin family that originated from algae expanded rapidly after the plants landed. The EXPA subfamily has more members and conservative evolution in angiosperms. *FtEXPA1*, *FtEXPA11*, *FtEXPA12*, *FtEXPA19* and *FtEXPA24* can respond to indole-3-acetic acid (IAA) signals and regulate fruit development. Our study provides a blueprint for improving the agronomic traits of Tartary buckwheat and a reference for defining the evolutionary history of the expansin family during plant transitions to land.

**Supplementary Information:**

The online version contains supplementary material available at 10.1186/s12864-021-07562-w.

## Background

Land plant radiation and colonization are important keystones in the evolutionary history of living organisms, which have created the ecological diversity on Earth that we see today. This transition was accompanied by complex and long biological evolution, which included morphological, physiological, and genetic changes, to cope with the terrestrial environment and its challenging conditions [1, 2]. The cell wall plays a key role in plant growth and development, material transport, pathogen resistance, cell division and differentiation, organ senescence and shedding. It also provides the necessary mechanical support for plant cells and the plasticity that is necessary for protection against external intrusion [3, 4]. The number and volume of plant cells always change dynamically, and both are regulated by cell wall plasticity during plant growth [5]. Studies have shown that the role of expansin proteins in the cell wall is critical to achieve this necessary plasticity [5]. Expansin is an important plant growth-regulating divisor that can realize the continuous assembly, remodeling and decomposition of cell walls [6]. It has significant functionality in many stages of plant growth and development [7], such as stem growth and internode elongation [8], fruit ripening [9], seed germination [10], control of flowering time and flower size [11], root growth [12] and leaf development [13].

Expansin proteins contain 250-275 amino acid residues [14] and consist of two conserved domains. The N-terminal conserved domain I (DPBB), which contains approximately 120-135 amino acids, is homologous to glycoside hydrolase family-45 (GH45). Previous studies have shown that there is no β-glucan sugar hydrolysis at the N-terminus of expansin proteins [15]. Another domain (domain II in the C-terminus) contains approximately 90-120 amino acids and has higher similarity with Group-II pollen allergen proteins (G2A family) and presumably is a polysaccharide binding domain (PLN) based on the polar residues on the surfaces of proteins and conserved aromatics [16]. To date, no other proteins containing domain II congeners have been found except for the G2A families [17]. A recent study established a 3D model of the FaEXPA2 protein that was involved in strawberry fruit softening and determined that FaEXPA2 formed a more stable complex with cellulose than other ligands via the different residues present in the open groove surface of its two domains [18]. Similarly, molecular dynamics showed that the FaEXPA5 protein is involved in strawberry fruit softening and can interact with ligands through the residues present in the open groove along the two domains [19]. Expansin proteins are cocoded by multiple gene families and are divided into the α-expansin (EXPA), β-expansin (EXPB), expansin-like A (EXLA), and expansin-like B (EXLB) subfamilies according to their phylogeny [20]. While EXLA and EXLB also possess two typical expansin protein domains, there is no experimental evidence that they also have the function of loosening cell walls [21]. Generally, EXPA is widely found in dicotyledonous and monocotyledonous plants, except non-*Poaceae*, while EXPB is mainly found in monocotyledonous plants [15]. Expansin proteins have been studied in many important species, including *Arabidopsis thaliana (A. thaliana)* [22], tea [23], *Solanum lycopersicum* [24], *Z. mays* [25], *Glycine max* [26], cotton [27] and wheat [28]. The EXPA subfamily was the first subfamily to be identified that contains cell wall-loosening proteins, which can quickly induce relaxation of the cell wall without lytic activity [29]. *AtEXPA7*, which is an EXPA family gene that is specifically expressed in root hair cells, was isolated from *A. thaliana*, and its biological function was detected by using RNA interference. The results showed that *AtEXPA7* played an indispensable role in root hair tip growth [30]. Overexpression of *AtEXPA2* promotes seed germination, while inhibition of its expression leads to a delay in seed germination [31]. Meanwhile, studies have shown that *AtEXPA2* may regulate seed germination through the GA signaling pathway [31]. The EXPB subfamily consists of two subgroups. Group-1 proteins are highly expressed in grass pollen [32] and can relax cell walls without destroying them [32]. Research on EXPA and EXPB is relatively deeper [33]. Recent reports have also confirmed the role of expansin proteins in fruit enlargement [34, 35], which provides new insights for improving crop agronomic traits.

Current agricultural studies are centered on the main staple crops, including rice, wheat and maize. However, this narrow research scope is not promising for providing systematic solutions to the challenges of food security and poverty [36]. Adding nutrient-rich pseudocereals to major cereals is a potential strategy to improve dietary diversity and provide alternative food stocks. Tartary buckwheat (*Fagopyrum tataricum*) is a versatile pseudocereal that is known as the golden crop [36]. It is also a traditional Chinese grain crop that is widely cultivated in China. Because of its strong environmental adaptability, it has become the main food source for people living in severe environments such as the southwest plateau of China [37]. Tartary buckwheat fruits are rich in starch, proteins, dietary fiber, vitamins and other nutrients [38]. In addition, the flavonoid contents in Tartary buckwheat are significantly higher than those of other foods, and proper intake can help organisms due to their antioxidant and anti-aging properties, as well as their ability to lower blood pressure and reduce the risk of arteriosclerosis [39]. Because of its important value in food and medicine, Tartary buckwheat has received more attention from breeding and genetic researchers in recent years. Some challenges in the breeding of Tartary buckwheat, such as increasing the dehulling efficiency of fruit, improving fruit quality, and increasing fruit size, remain to be solved [40].

Considering the important role of expansin proteins in plant development and adaptation to complex terrestrial environments, we identified 323 expansin proteins in 12 genomes from algae to angiosperms. We studied these proteins by performing phylogenetic analysis, gene structure and motif composition analysis, cis-acting element identification of promoter regions, and gene duplication. We also analyzed the origin and evolution of expansin proteins in representative plants during plant terrestrialization. More importantly, we identified five candidate genes from the EXPA subfamily that may improve the important agronomic traits of Tartary buckwheat, which was accomplished by combining the expression of 37 genes in different tissues and organs, especially in the important stages of fruit development. In summary, our study identified the FtEXP gene family for the first time. The conservation and evolution of this species in the process of plant landing are discussed, and its potential regulatory roles in fruit development and hormone response are determined, which provides new insights for Tartary buckwheat breeding.

## Results

### Global identification and evolution of Expansin proteins from algae to land plants

**Fig. 1:**
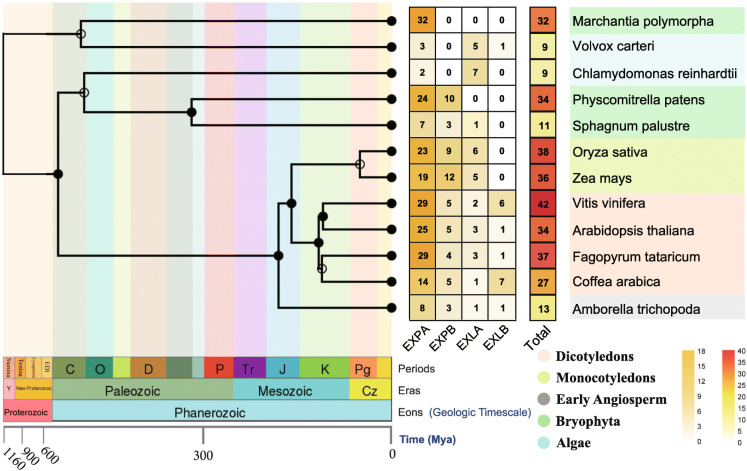
Phylogeny and diversity of expansin proteins in 12 species. A species tree was constructed using the online software TIMETREE (http://www.timetree.org/). The number of members in different subfamilies is expressed by a color scale. The blue, green, gray, light green and orange colors represent algae, Bryophyta, early angiosperms, monocotyledons and dicotyledons, respectively

To further understand the evolutionary history of expansin during plant transitions to land, we identified 323 *expansin* genes by using BLAST and profile HMM searches of two algae (*Chlamydomonas reinhardtii* and *Volvox carteri*); three bryophytes (*Marchantia polymorpha*, *Physcomitrella patens* and *Sphagnum palustre*); early angiosperms (*Amborella trichopoda*); two monocotyledons (*Oryza sativa* and *Zea mays*) and four dicotyledons (*F. tataricum*, *Arabidopsis*, *Vitis vinifera* and *Coffea arabica*) (Fig. 1, Table S1). We divided the expansin family into four subfamilies (EXPA, EXPB, EXLA and EXLB) according to the distribution and structural characteristics of the Arabidopsis EXP (AtEXP) members [20] (Fig. 1, Table S2).

Furthermore, the numbers of expansins in each subgroup of these species were investigated (Fig. 1, Table S1). There were fewer members of the algae EXPA subfamily and more members of the EXLB subfamily, which was in sharp contrast to higher plants (Fig. 1). Interestingly, up to 32 members of the EXPA subfamily were found in *M. polymorpha*, while other subfamily members were not found, which shows that the EXPA subfamily began to expand as the plant made the transition to land. In monocotyledon species, EXPB was the larger subfamily, while EXPA was the larger subfamily in dicotyledons. EXLB was present only in early angiosperms and dicotyledons but not in other plants except for *V. carteri*, and EXPA arose early in the evolution of bryophytes and was conserved across land plants (Fig. 1).

### Analysis of phylogeny and evolution suggests that the FtEXPA subfamily has rich members and special structures

**Fig. 2:**
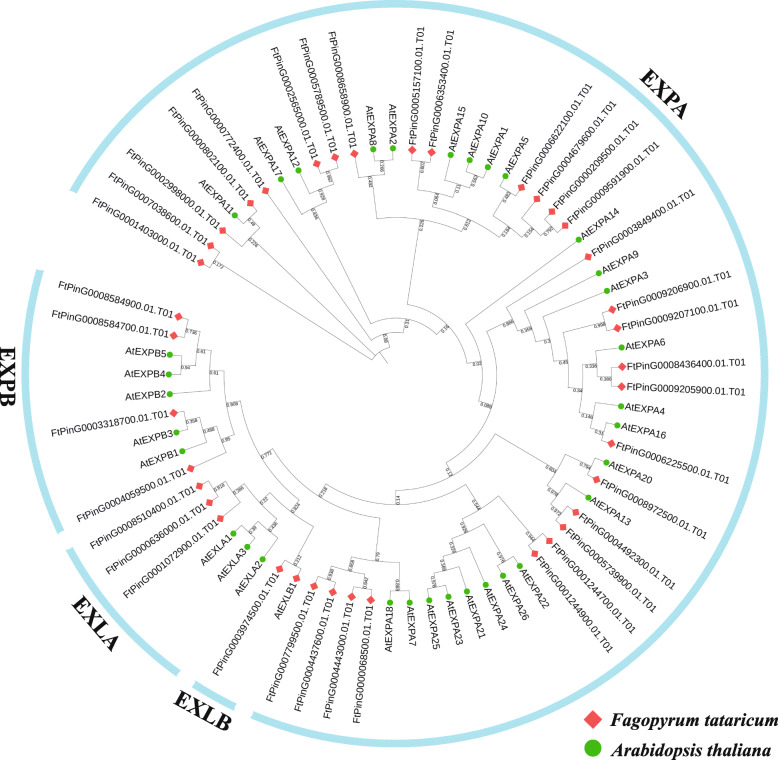
Phylogenetic tree that represents the relationships among 37 *expansin* genes of Tartary buckwheat and 34 *expansin* genes of *A. thaliana*. The phylogenetic tree of the expansin protein sequences of Tartary buckwheat and *A. thaliana* was constructed with Mega 7.0 by the maximum likelihood method and was visualized by the online tool Interactive Tree Of Life (iTOL) (http://itol2.embl.de/). The genes in Tartary buckwheat are marked in red diamond, while those in *A. thaliana* are marked in green circle

We identified 37 expansin proteins in the Tartary buckwheat genome and assembled the basic information for these genes, such as Mw, PI, subcellular localization, CDS and protein sequence (Table S3-4). Based on the multiple sequence alignment of 37 FtEXP proteins and 34 *A. thaliana* expansin proteins, we reconstructed a maximum likelihood phylogenetic tree to explore the evolutionary relationships of expansin proteins in Tartary buckwheat (Fig. 2). The number of genes in different subfamilies varies. The EXLB subfamily has the lowest number of members (only one gene), and the EXPA subfamily has the largest number of genes (Fig. 2). The number of expansin proteins in each subfamily of Tartary buckwheat is very close to that in *A. thaliana*.

**Fig. 3:**
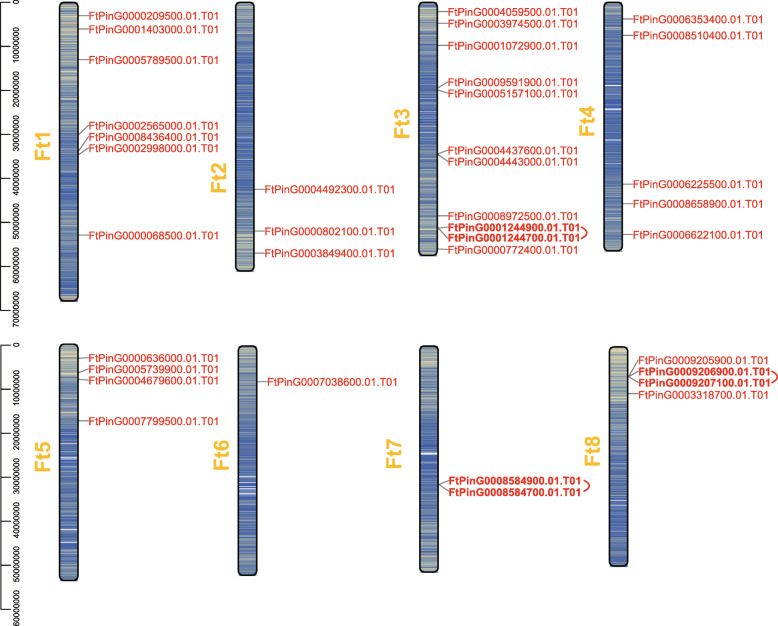
Schematic representations of the chromosomal distributions of the Tartary buckwheat *expansin* genes. Gff files and sequencing files were used to obtain chromosome localization information of FtEXPs and visualized by TBtools v1.082. The chromosome number is indicated to the left of each chromosome. The red lines behind the genes indicate that they are pairs of tandem duplication genes

Furthermore, we mapped all *FtEXPs* to 8 chromosomes, based on physical location information from the Tartary buckwheat genome generic feature format (Gff) data (Fig. 3). The 37 *FtEXPs* are unevenly distributed on 8 chromosomes. Most genes are on chromosome 3 (eleven genes), and the fewest are on chromosome 6 (only one gene). The genes on chromosome 7 and chromosome 8 are also less distributed, but each chromosome has a tandem duplicate region. Multiple *FtEXPs* are distributed on chromosomes 1, 3 and 4, but only one pair of tandemly duplicated genes was detected on chromosome 3 (Fig. 3). Two pairs of EXPA subfamily genes (*FtPinG0001244700.01*-*FtPinG0001244900.01* and *FtPinG0009206900.01- FtPinG0009207100.01*) from chromosomes 3 and 8 are tandem duplications, which may have contributed to the expansion of the EXPA subfamily to some extent. In addition, 37 FtEXPs were renamed according to their subfamilies and chromosomal distributions (Table S3).

**Fig. 4:**
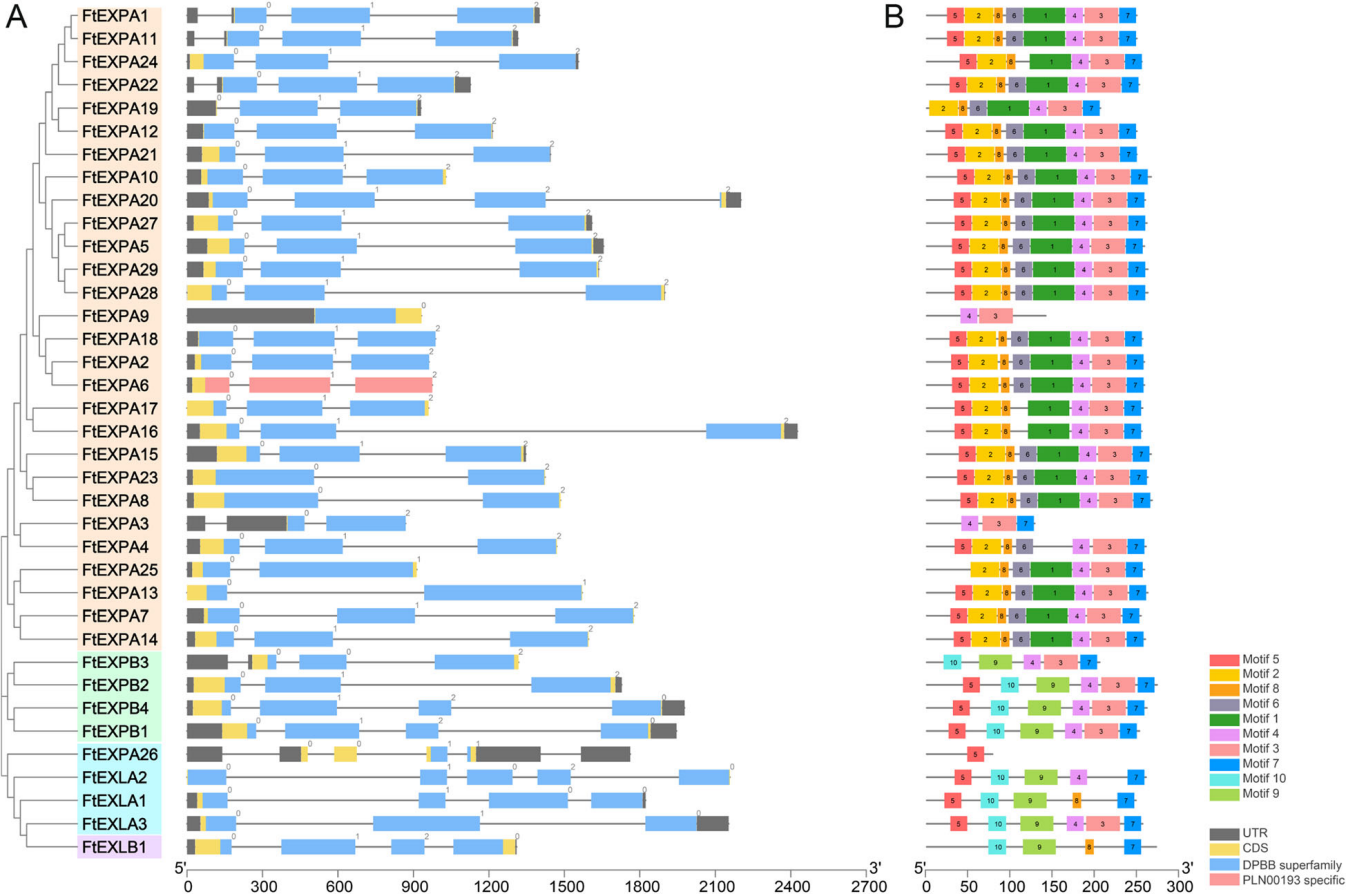
Phylogenetic relationships, gene structures, and architectures of the conserved protein motifs of the *expansin* genes from Tartary buckwheat. **a** The phylogenetic tree was constructed based on the full-length sequences of Tartary buckwheat expansin proteins using MEGA 7.0 and was visualized by the online tool Interactive Tree Of Life (iTOL) (http://itol2.embl.de/). Orange represents the EXPA subfamily gene, green represents the EXPB subfamily gene, blue represents the EXLA subfamily gene, and purple represents the EXLB subfamily gene. Prediction of the exon-intron structures of Tartary buckwheat expansin genes was performed using the online Gene Structure Display Service 2.0 (http://gsds.gao-lab.org/) and was visualized by TBtools v1.082. Gray boxes indicate untranslated 5′- and 3′-regions, and black lines indicate introns. The number indicates the phases of the corresponding introns. **b** The motif compositions of the Tartary buckwheat expansin proteins. The conserved motifs of expansin proteins were determined by the MEME online program (http://meme-suite.org/tools/meme) and were visualized by TBtools v1.082. The motifs, numbered 1-10, are displayed in different colored boxes. The sequence information for each motif is provided in Table S5. Protein lengths can be estimated using the scale at the bottom

We also investigated the exon-intron organizations of all identified *FtEXPs* for a deeper understanding of the evolution of this family in Tartary buckwheat (Fig. 4a). Among 37 *FtEXPs*, the number of introns ranged from 0 to 3, and most members of the EXPA subfamily contained 2 introns. Notably, the structure of several members of the EXPA subfamily is special; for example, only FtEXPA6 (*FtPinG0002998000.01*) contains a PLN domain, and FtEXPA26 (*FtPinG0007038600.01*) contains five introns, while its exon length is significantly different from those of the other genes (Fig. 4a). Analysis of the motifs was performed through the online MEME software to further study the characteristic regions of the FtEXP proteins (Fig. 4b). Most members of the EXPA subfamily contain motifs 1 to 8, while most members of the other subfamilies contain motifs 3, 4, 7, 9 and 10 (Fig. 4b). Notably, some genes contain very few motifs; for example, FtEXPA26 (*FtPinG0007038600.01*) contains only motif 5, while FtEXPA9 (*FtPinG0000802100.01*) contains only motifs 3 and 4. Overall, most genes from the same subfamily have similar motif compositions, and the expansin proteins of the other 11 plants also have conserved domains and general characteristics (Fig. S1-S2, Table S5).

Environmental stress can profoundly affect the growth and development of plants [41]. We analyzed the cis-acting elements of 37 *FtEXP* promoter regions by using PlantCARE software to investigate their responses to the environment. Three environmentally responsive elements were detected, including light-, low temperature- and defense stress resistance-responsive elements, and they were widespread in 37 *FtEXPs* (Fig. S3). Most hormone-responsive elements (MeJA, auxin, abscisic acid and gibberellin) were also widely distributed in all *FtEXPs*, except the salicylic acid-responsive elements (Fig. S3). Salicylic acid-responsive elements exist only in the EXPA subfamily, and such responsive elements that are related to plant disease resistance [42] and drought tolerance [43] have attracted our attention.

### Gene duplication and evolutionary analysis of Expansin gene families in representative species

**Fig. 5:**
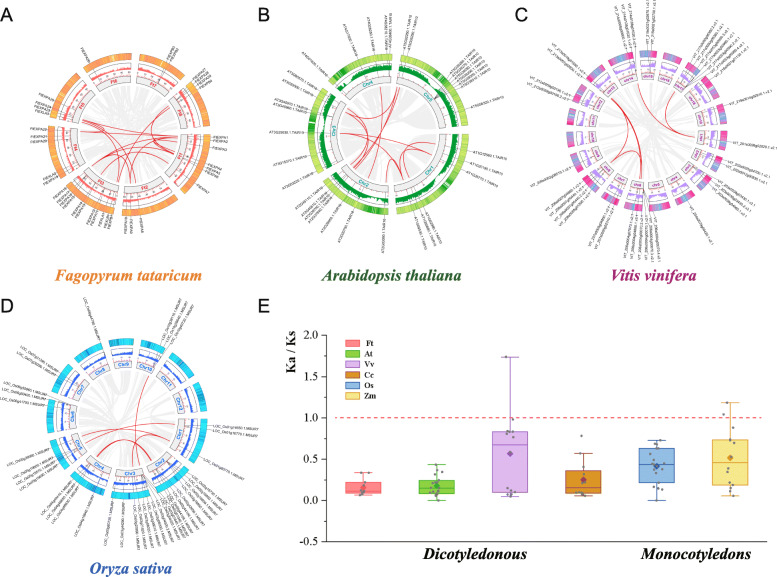
Schematic representations of the interchromosomal relationships of the *expansin* genes from different plants. **a** Analysis of the interchromosomal relationships of the *expansin* genes from Tartary buckwheat was conducted by using multiple collinear scanning toolkits (MCScanX) and was visualized by TBtools v1.082. Gray lines in the background indicate collinear blocks within the Tartary buckwheat genome, while red lines highlight syntenic *expansin* gene pairs. **b** Analysis of the interchromosomal relationships of the *expansin* genes from *A. thaliana* was conducted by using multiple collinear scanning toolkits (MCScanX), and was visualized by TBtools v1.082. Gray lines in the background indicate collinear blocks within the *A. thaliana* genome, while red lines highlight syntenic *expansin* gene pairs. **c** Analysis of the interchromosomal relationships of the *expansin* genes from *Vitis vinifera* was conducted by using multiple collinear scanning toolkits (MCScanX), and was visualized by TBtools v1.082. Gray lines in the background indicate collinear blocks within the *Vitis vinifera* genome, while red lines highlight syntenic *expansin* gene pairs. **d** Analysis of the interchromosomal relationships of the *expansin* genes from *Oryza sativa* was conducted by using multiple collinear scanning toolkits (MCScanX), and was visualized by TBtools v1.082. Gray lines in the background indicate collinear blocks within the *Oryza sativa* genome, while red lines highlight syntenic *expansin* gene pairs. **e** KaKs_Calculator 2.0 was used to calculate the synonymous (Ks) and nonsynonymous (Ka) substitutions of each homologous expansin gene pair and their ratios (Ka/Ks). The results were visualized using TBtools v1.082

Gene duplication that arises from tandem duplication or during polyploidization and segmental duplication associated with replication is a major factor causing family expansion. For a deeper understanding of the evolution of expansin homologous copy genes, we conducted a syntenic analysis of the expansin proteins from four dicotyledons (*F. tataricum*, *Arabidopsis*, *C. arabica* and *V. vinifera*) and two monocotyledonous plants (*O. sativa* and *Z. mays*). We detected 14 pairs of segmental duplications on different chromosomes of Tartary buckwheat (Fig. 5a). Most segmental duplication genes also came from the EXPA subfamily (*FtPinG0000209500.01*, *FtPinG0002998000.01*, *FtPinG0000802100.01*, *FtPinG0006622100.01* and *FtPinG0004679600.01*), which could be another important reason why the EXPA subfamily expanded within species. The results also showed that different pairs of segmental duplication EXP gene pairs were found in the genomes of Arabidopsis (22 pairs), *V. vinifera* (6 pairs), and *O. sativa* (6 pairs) (Fig. 5b-d). To explore the different selective constraints of the duplicated *FtEXP* pairs, we calculated the Ks values and Ka/Ks ratios of each homologous gene pair between Tartary buckwheat and other terrestrial plants (Table S6). The Ka/Ks values of the majority of expansin homologous gene pairs were less than 1, especially for the EXPA subfamily, which indicated that expansin genes are highly conserved in evolution and can be important for plant growth and development (Fig. 5e, Table S6).

**Fig. 6:**
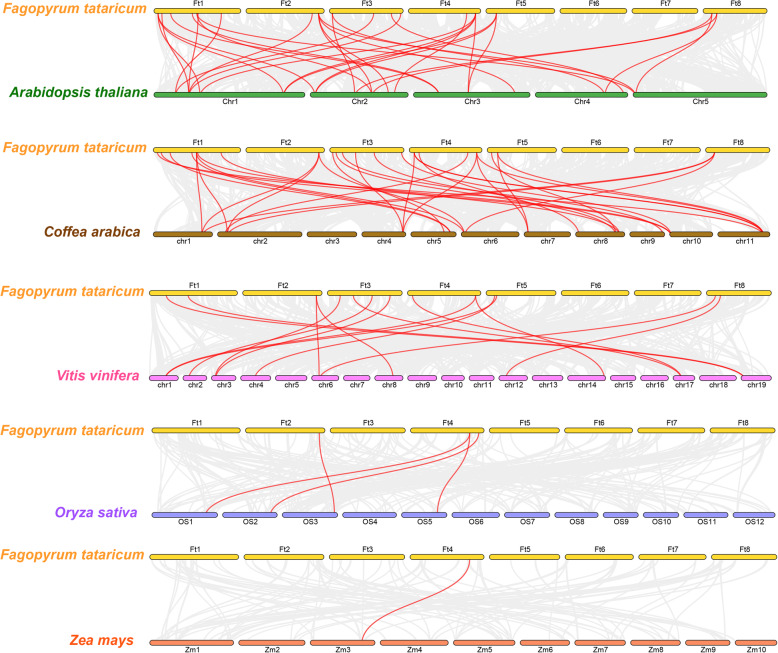
Synteny analyses between the expansin genes of Tartary buckwheat and five angiosperms. The syntenic relationships among the expansin genes of Tartary buckwheat and five angiosperms were visualized by using Dual Synteny Plotter software. The results were visualized using TBtools v1.082. Gray lines in the background indicate collinear blocks within the Tartary buckwheat genome and other plant genomes, while red lines highlight syntenic expansin gene pairs

Previous reports have shown that synteny occurs not only within species; synteny genes between species are often another channel for the rapid evolution of gene families and are prone to copy genes with similar functions [44]. Therefore, we further investigated syntenic genes that are homologous to Tartary buckwheat expansins in representative plants. Syntenic expansin gene pairs are widely found among Tartary buckwheat and *Arabidopsis* (32 homologous gene pairs), *C. arabica* (32 homologous gene pairs), *V. vinifera* (15 homologous gene pairs), *O. sativa* (5 homologous gene pairs), and *Z. mays* (only 1 homologous gene pair) (Fig. 6, Table S7).

### Differential expression of EXPA subfamily genes in different tissues of Tartary buckwheat

Many reports have shown that expansin proteins are closely related to plant growth and development, especially the fruit development of angiosperms; examples include *A. thaliana* [45], wheat [46], rice [47], tomatoes [48], and tobacco [49]. Therefore, we detected the expression of 37 *FtEXPs* in different tissues of Tartary buckwheat by quantitative real-time polymerase chain reaction (qRT-PCR).

**Fig. 7:**
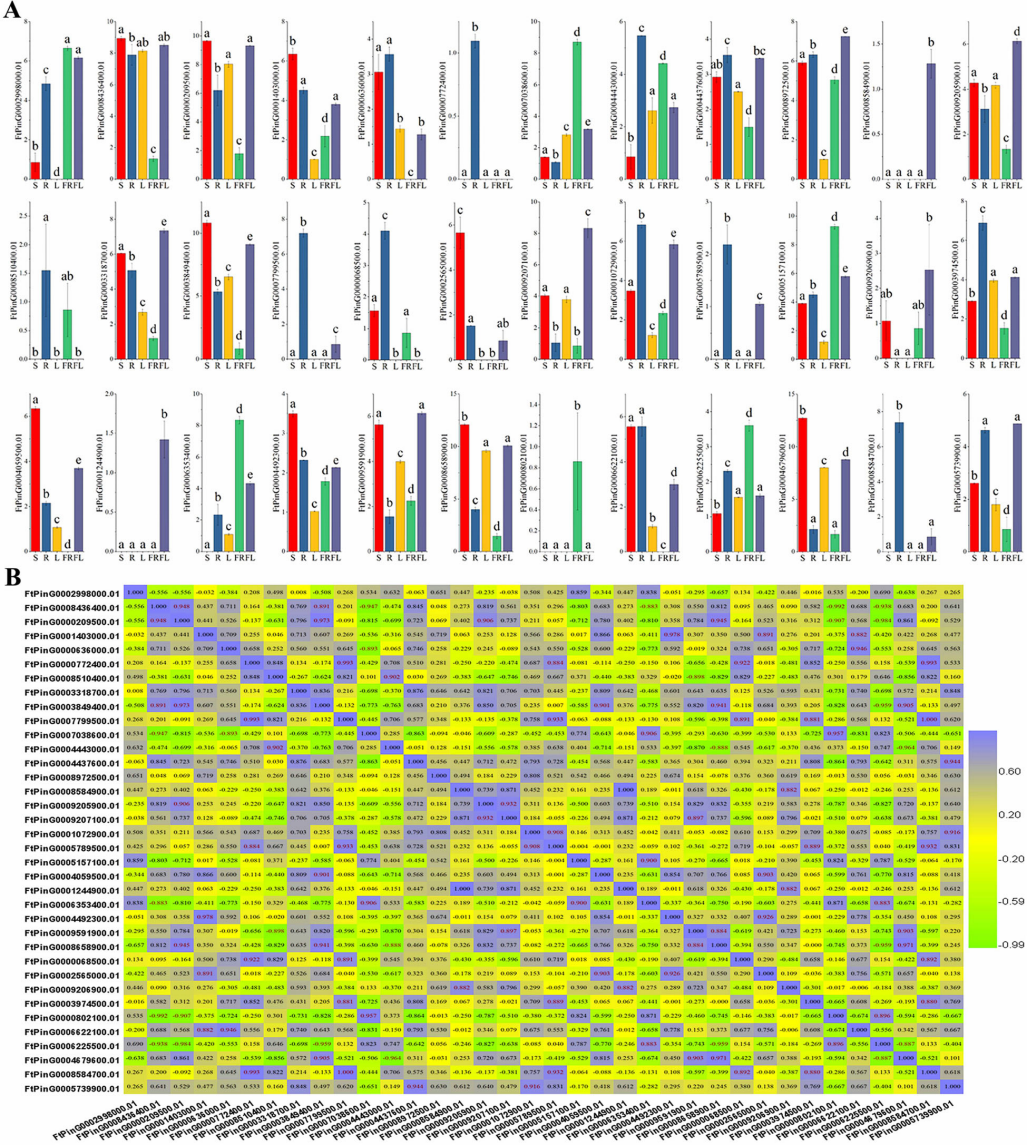
Tissue-specific gene expression of Tartary buckwheat *expansin* genes and the correlation between the gene expression patterns of *FtEXPs*. **a** The expression patterns of Tartary buckwheat *expansin* genes in flower (FL), leaf (L), root (R), stem (S) and fruit (FR) were examined by qRT-PCR. Three biological replicates were performed, and three technical replicates were performed for each biological replicate. Error bars were obtained from nine measurements. Lowercase letter(s) above the bars indicate significant differences (α = 0.05, LSD) among different tissues. The results were visualized using GraphPad Prism 7.04. **b** The correlation of expression patterns of Tartary buckwheat expansin genes in different tissues was visualized by TBTools v1.082. Positive number: positively correlated; negative number: negatively correlated. The red numbers indicate a significant correlation at the 0.05 level

The histograms show that all *FtEXPs* were expressed except *FtPinG0001244700.01*. Twenty genes exhibited expression in each tissue. There were some tissue-specific genes, of which *FtPinG0000772400.01* was a specific gene that was expressed only in roots, and *FtPinG0008584900.01* and *FtPinG0001244900.01* were specific genes that were expressed only in flowers (Fig. 7a). Among the 36 genes, 12 genes had the highest expression levels in roots, and 5 genes had the highest expression levels in stems. Interestingly, we found six *FtEXPs* with special expression in fruit, including five genes (*FtPinG0002998000.01*, *FtPinG0007038600.01*, *FtPinG0005157100.01*, *FtPinG0006353400.01* and *FtPinG0006225500.01*) with significantly higher expression than in other tissues, and one gene (*FtPinG0000802100.01*) that was expressed only in fruit. The six special genes were all from the EXLA subfamily, although the *FtPinG0000802100.01* expression was relatively low. Members of the EXPA subfamily are generally involved in the regulation of plant fruit development, which has been fully confirmed in previous studies [50].

Moreover, we also provided the correlations among the expression levels of each gene. We can see from the correlation analysis of the 36 genes expressed in different tissues that there were positive correlations among the expression profiles of most genes, especially the six fruit-specific genes mentioned earlier, all of which were significantly positively correlated (Fig. 7b).

### Expression patterns of EXPA subfamily members were different in the three important periods of fruit development

**Fig. 8:**
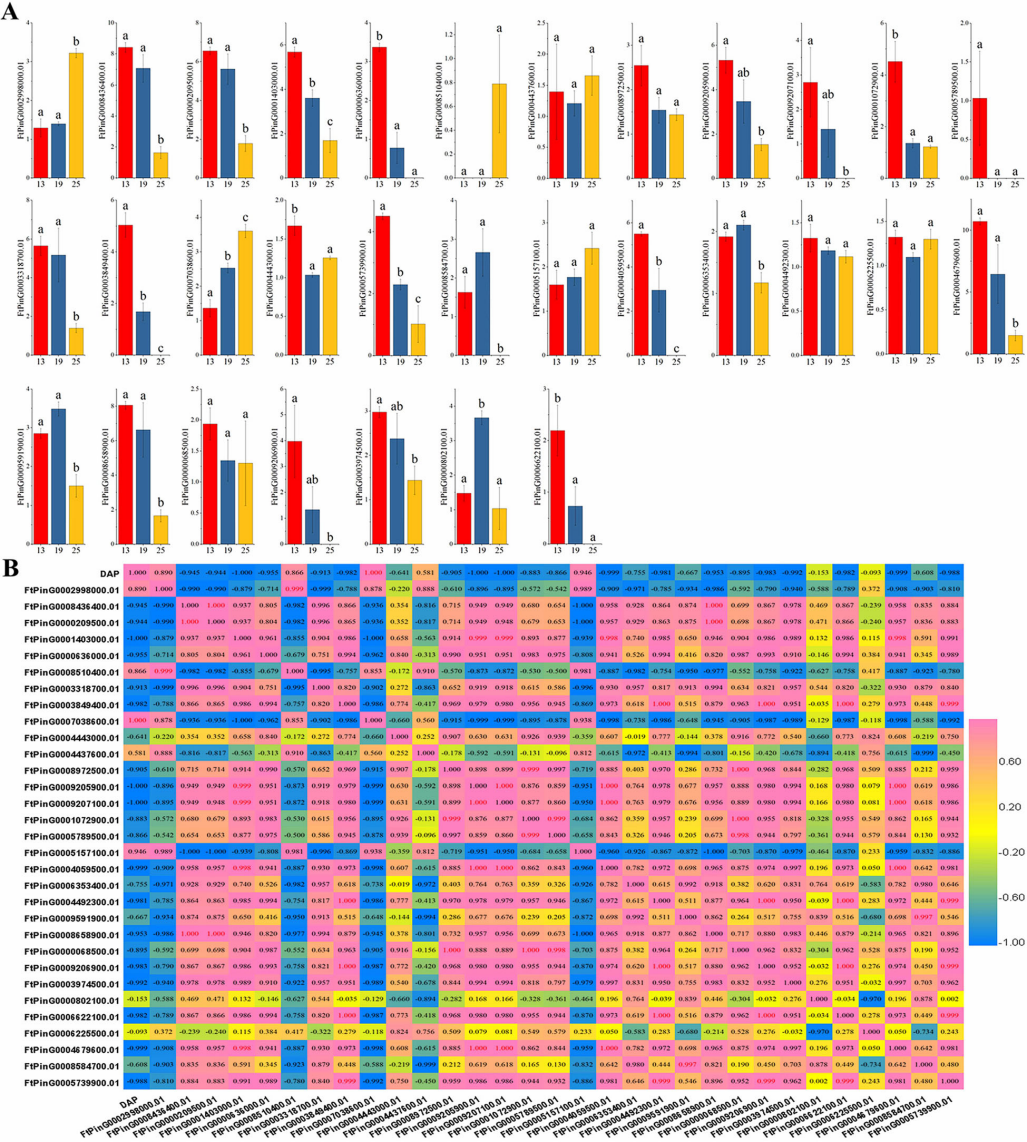
Gene expression of Tartary buckwheat *expansin* genes during fruit development and the correlation between the gene expression patterns of *FtEXPs* during fruit development. **a** The expression patterns of Tartary buckwheat *FtEXPs* in the fruit developmental stages were examined using a qRT-PCR assay. Three biological replicates were performed, and three technical replicates were performed for each biological replicate. Error bars were obtained from nine measurements. Lowercase letter(s) above the bars indicate significant differences (α = 0.05, LSD) among fruits at different developmental stages. The results were visualized using GraphPad Prism 7.04. **b** The correlation of expression patterns of Tartary buckwheat *expansin* genes in different fruit developmental stages was visualized by TBTools v1.082. Positive number: positively correlated; negative number: negatively correlated. Red numbers indicate a significant correlation at the 0.05 level

In the preliminary study, we divided Tartary buckwheat into five stages from anthesis to maturation according to embryonic development morphology, among which the green fruit stage (13 DAP), discoloration stage (19 DAP) and initial maturity stage (25 DAP) were the three most important developmental stages [51]. To screen the potential *FtEXPs* regulating fruit development, we determined the expression of 31 *FtEXPs* during the three most important fruit development stages (13 DAP, 19 DAP and 25 DAP) by qRT-PCR. The results showed that the expression of 4 genes increased gradually at 13 DAP, 19 DAP and 25 DAP, including three genes from the EXPA subfamily (*FtPinG0002998000.01, FtPinG0007038600.01* and *FtPinG0005157100.01*) and one gene from EXPB (*FtPinG0008584700.01*), which was not expressed at 25 DAP. In addition, among the genes that were expressed in all three periods, six genes experienced both upregulation and downregulation. Three EXPA subfamily genes that were specifically expressed in fruit (*FtPinG0006353400.01*, *FtPinG0006255000.01* and *FtPinG0000802100.01*) were also within the range (Fig. 8a).

From the correlation study of 31 *FtEXP* expression levels in fruits at different developmental stages, it can be seen that some genes showed significant negative correlations with nearly all genes, such as *FtPinG0002998000.01*, *FtPinG0008510400.01*, *FtPinG0007038600.01*, *FtPinG0006255000.01* and *FtPinG0005157100.01* (Fig. 8b). High gene expression levels in a certain tissue or development phase indicate that the gene may perform certain actions during the growth and development of this tissue, while some negatively related genes may have developed differences in function. In general, there were close correlations among those genes of the EXPA family that were highly expressed in fruit (Fig. 8b). At all stages of fruit development, the variation trends of EXPA subfamily expression were not exactly the same, and negative correlations of some genes were obvious (Fig. 8).

### Five genes from the EXPA subfamily were identified to regulate fruit development by responding to Indole-3-acetic acid (IAA) signals

**Fig. 9:**
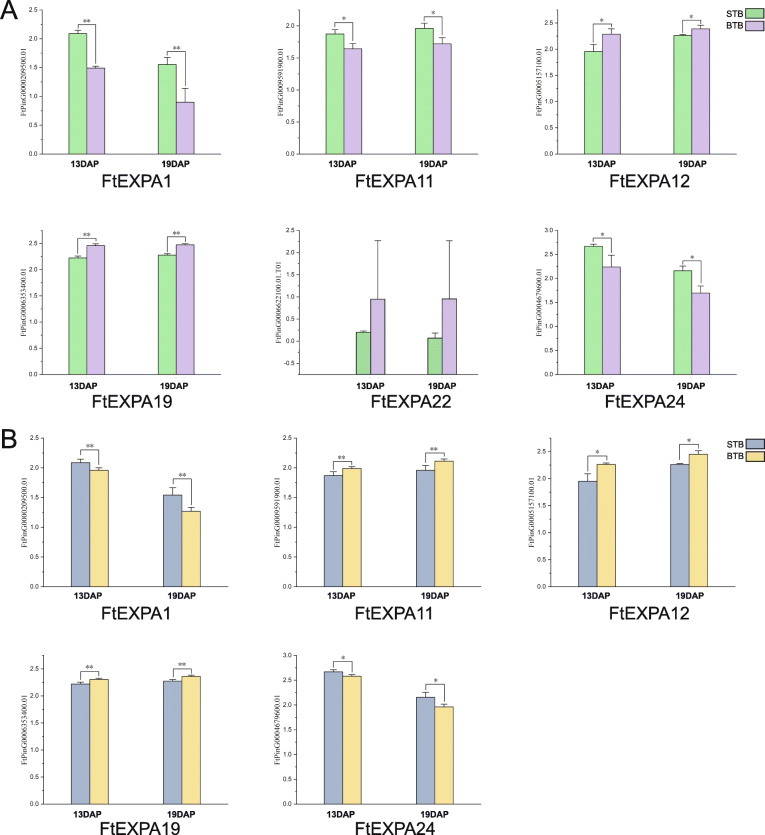
Gene expression of six *FtEXPA* genes in the BTB and STB at 13 DAP and 19 DAP and the expression patterns of five *FtEXPA* genes from 13 DAP and 19 DAP of the STB with IAA treatment. **a** The expression patterns of six *FtEXPA* genes in BTB and STB at 13 DAP and 19 DAP were examined using a qRT-PCR assay. Three biological replicates were performed, and three technical replicates were performed for each biological replicate. Error bars were obtained from nine measurements. Asterisk above the bars indicate significant differences (*P < 0.05; **P < 0.01) between the BTB and STB fruits. The results were visualized using GraphPad Prism 7.04. **b** The expression patterns of five *FtEXPA* genes in STB at 13 DAP and 19 DAP with IAA treatment were examined using a qRT-PCR assay. Three biological replicates were performed, and three technical replicates were performed for each biological repeat. Error bars were obtained from nine measurements. Asterisk above the bars indicate significant differences (*P < 0.05; **P < 0.01) between mock and IAA. The results were visualized using GraphPad Prism 7.04

Studies have shown that overexpression of *AtEXPA10* in tobacco affects the size of reproductive organs, while overexpression of *NtEXPA5* in tobacco increases the size of tobacco leaf and stem cells [49]. To further screen expansin proteins that potentially regulate fruit size, six genes with the highest homology to the previously reported *AtEXP10* (*AT1G26770.2*) were selected in the phylogenetic tree (Fig. 1). The expression of these six *FtEXPA* genes in BTB fruit and STB fruit was determined (Fig. 9a). The results showed that there were significant differences in the expression of the other five genes except *FtPinG0006622100.01* in STB and BTB fruits. Among them, the expression of three genes (*FtPinG0009591900.01*, *FtPinG0000209500.01* and *FtPinG0004679600.01*) in STB fruits was higher than that in BTB fruits, and the expression of the other two genes *(FtPinG0006353400.01* and *FtPinG0005157100.01*) in BTB fruits was higher than that in STB fruits. Auxin plays an indispensable role in the expansion of plant organs [52]. The Tartary buckwheat fruit size reached a maximum at 19 DAP, and the auxin content in Tartary buckwheat fruits increased gradually from 13 DAP to 19 DAP [51]. *FtPinG0006353400.01* and *FtPinG0005157100.01* were both highly expressed in BTB fruits, and their expression levels in the fruit development stage were consistent with the changes in the auxin content in fruits. Through the above results, we found genes that were differentially expressed in the STB and BTB fruits, which further narrowed the candidate range of fruit size-regulating genes.

The studies above showed that several EXPA subfamily genes (*FtPinG0009591900.01*, *FtPinG0000209500.01*, *FtPinG0006353400.01*, *FtPinG0004679600.01* and *FtPinG0005157100.01*) were highly expressed in both the STB and BTB fruits, and there were significant differences in the expression of these genes between the two types of fruits. A previous study showed that the fruit weights of STB increased with exogenous auxin treatment [51]. Therefore, we measured the expression of these five genes in STB fruits under IAA treatment to further screen potential genes for regulating fruit size. The results showed that the expression of three genes (*FtPinG0009591900.01*, *FtPinG0006353400.01* and *FtPinG0005157100.01*) increased and that the expression of two genes (*FtPinG0004679600.01* and *FtPinG0000209500.01*) decreased with IAA treatment (Fig. 9b).

## Discussion

Expansin proteins can regulate many plant growth and development processes by participating in the synthetic modification of cell walls. As a result, the expansin gene family is valuable for plant growth and development [5]. In addition, expansin proteins are not found in animal and fungal species, while they are widespread in plants ranging from algae to higher plants, which also makes them of fascinating significance for studying the terrestrial evolution of plants. Based on our phylogenetic research, expansin proteins have at least one DPBB conserved domain in both algae and higher plants (Fig. S1). The EXPA subfamily has the largest number of members and was already present in algae. The EXPB subfamily originated later and had a smaller number of members. The surprising number of EXPA subfamily members in the basal terrestrial moss (*M. polymorpha*) may suggest that the family’s genes expanded as plants transitioned to land (Fig. 1). Abundant gene duplication events drove the expansion of expansin families in angiosperms, and the Ka/Ks values of most homologous EXP gene pairs in both monocotyledonous and dicotyledonous plants were all obviously less than 1, which indicates that the expansin family received purification options after plant terrestrialization (Fig. 5).

Evolutionary analysis of expansin families provides valuable insight into the regulation of important agronomic traits in Tartary buckwheat genetics and breeding. Our first key finding is that the Tartary buckwheat EXPA subfamily expanded after gene duplication, and the evolution direction of the members varied. Compared with the other three subfamilies, the EXPA subfamily has the most members, as has been reported for other plants [46]. It has been reported that segmental duplication events contribute to gene expansion [53]. This conclusion is further supported by the finding that most members of the Tartary buckwheat EXPA subfamily undergo tandem and segmental duplication (Fig. 3, Fig. 5a). Studies have shown that gene duplication events can trigger family expansion [54], and the genes that undergo duplication have three evolutionary outcomes: maintaining the original conserved function, generating new functions or forming pseudogenes [55]. *FtPinG0001244900.01*-*FtPinG0001244700.01* and *FtPinG0009206900.01*-*FtPinG0009207100.01* are two pairs of tandem duplicated genes located on chromosomes 3 and 8 (Fig. 3), all of which are from the EXPA subfamily. Among them, the expression of *FtPinG0009206900.01* and *FtPinG0009207100.01* was obviously different (Fig. 8a), which indicates that they may have evolved into two genes with different functions. Furthermore, it was found that the motif compositions of these two genes were consistent, and it was suggested that the difference in function may originate from the difference in cis-acting elements in the promoter region (Fig. 4, Fig. S3). In contrast, *FtPinG0001244900.01* is only expressed in flowers, and *FtPinG0001244700.01* had no expression (Fig. 8a), which indicated that *FtPinG0001244700.01* may have become a pseudogene after duplication events. Whether pseudogenes are functional is an unresolved issue, and some argue that pseudogenes provide binding sites for transcription factors [56]. At least it is certain that these two pairs of tandem duplication genes from the same subfamily evolved in different directions after duplication occurred.

Another important finding is that duplication and loss of expansin proteins are common in representative angiosperms, and the conservative traits that were acquired from ancestors are favored by natural selection. This study found that two subfamilies (EXPA and EXPB) are conserved in representative angiosperms, which implies that they may already have existed in the common ancestor of angiosperms and were preserved after species differentiation. Studies have shown that certain plants that adapted to the land environment have lost some expansin proteins during evolution [57], which can provide a possible explanation for the larger number of EXLA subfamilies in algae, while few other species exist (Fig. 1). It has been reported that the expansion and contraction of gene families is a manifestation of the rapid adaptation of organisms to the environment [58]. However, new genes that formed with family expansion often faced harsh natural selection [59]. The Ka/Ks values of *FtEXPs* were also all obviously less than 1 (Table S6), which indicated that the *FtEXPs* were purified during evolution. Specifically, although new genes were generated through evolutionary processes, the primitive functional genes that originated from ancestors were more conducive to adaptation to the environment [60]. Furthermore, EXPA subfamily genes may also have experienced motif loss and functional alternatives during evolution. Within the subfamily, motifs 5 and 10 do not usually coexist. One possible explanation is that the functions of motif 5 and motif 10 are redundant, and only one of them was preserved after a long period of evolution. Studies have also shown that the extra lost motif 1 (DPBB domain) is predicted to contain a signal peptide sequence (Fig. 4, Table S5) and can bind to cellulose [61], which may be a key factor for EXLA and EXLB not having cell wall-loosening functions.

Unfortunately, studies have shown that expansin proteins inevitably have negative effects on immune function while increasing fruit yield. Cell wall loosening is the direct cause of fruit organ enlargement [5], but expansion of the cell wall also leads to an increase in the gaps between cells, which may make plants vulnerable to external pathogens. Overexpression of indole-3-acetic acid–amido synthetase GH3-8 in rice induces IAA accumulation and then inhibits expansin protein expression [62]. Previous experimental evidence has confirmed that the disease resistance of rice was significantly enhanced, but its development was delayed [62]. This appears to indicate that we must make a choice between fruit enlargement and pathogen defense or other means to compensate for the lack of immune capacity that is caused by high expansin protein expression. Of the two FtEXPA subfamily genes (*FtPinG0004679600.01* and *FtPinG0000209500.01*) that were significantly downregulated after exogenous IAA treatment in our study (Fig. 9b), we suggest that the response of these two genes under IAA treatment may be similar to those mentioned in reports on rice [62]. For future studies, we should integrate other developmental, evolutionary, and ecological aspects to improve plant disease resistance to compensate for deficiencies in immune function after expansin protein expression.

## Conclusions

Collectively, our research not only identified all expansin family members in the 12 representative plants during terrestrial processes, but also from the perspective of evolution, a blueprint was drawn for the selection and cultivation of the important agronomic traits of Tartary buckwheat fruit development. The expansin family originated from early algae that expanded rapidly after plant terrestrialization. EXPA subfamily members that are dependent on gene duplication expansion provide insights into Tartary buckwheat genetics and breeding. Notably, we identified five key candidate genes from the EXPA subfamily that could potentially regulate fruit size. Identification of target genes through evolutionary analyses at the whole-genome level can provide new insights for crop breeding. Our results will also contribute to improving the important agronomic properties of Tartary buckwheat. In addition, the research highlighted a new challenge regarding balancing the tradeoff between high yield and disease resistance of fruit, which provides an idea for future breeding.

## Methods

### Plant genome sequence acquisition and identification of the Expansin gene family

The Arabidopsis genome sequence was acquired from the TAIR database (https://www.arabidopsis.org/). The Tartary buckwheat genome was obtained from the Tartary buckwheat Genome Project (http://www.mbkbase.org/Pinku1) [40]. Other plant genome sequences (*M. polymorpha*, *V. carteri*, *C. reinhardtii*, *P. patens*, *S. palustre*, *O. sativa*, *Z. mays*, *V. vinifera*, *C. arabica* and *A. trichopoda*) were downloaded from the Phytozome database (http://www.phytozome.net/). The hidden Markov model (HMM) profiles of two domains (PF03330 and PF01357) were obtained from the Pfam protein family database (http://pfam.sanger.ac.uk/). HMMER3.0 was used to identify *EXPs* from the genomes. SMART [63], Pfam [64] and InterPro [65] were used to verify whether the identified genes had conserved domains and to remove the genes without conserved domains. Then, the expansin proteins identified above were BLASTp searched in NCBI to analyze whether they were part of the expansin family. We identified all expansin proteins from the genomes of 12 plants by using the above methods. Information on the isoelectric point (PI) and molecular weight (Mw) was acquired from the ExPASy website (https://web.expasy.org/protparam/). The subcellular localizations of the FtEXP proteins were predicted with CELLO (http://cello.life.nctu.edu.tw/).

### Phylogenetic analyses, chromosomal location, intron-exon structure, motif composition and Cis-acting element analysis

TBtools v1.082 was used to extract CDSs from all plant genomes and translate them into protein sequences [66]. The expansin protein sequences from different plants were aligned by using the Clustalx1.81 program [67]. The Clustalx1.81 parameters were defined as follows: in pairwise alignment, the gap opening penalty was 10, and the gap extension penalty was 0.1; in multiple alignments, the gap opening penalty was also 10, but the gap extension penalty was 0.2. The phylogenetic tree of the expansin protein sequences in different plants was constructed with Mega 7.0 by the maximum likelihood method and 1000 bootstrap replications. The phylogenetic tree of Tartary buckwheat and *A. thaliana* was established to define the grouping of *FtEXPs* and was constructed by the above method. After that, the Clustalx1.81 program was used to align the expansin protein sequences of Tartary buckwheat and *A. thaliana*. Predictions of intron structures with expansin DNA sequences were performed by using the online Gene Structure Display Service 2.0 (http://gsds.gao-lab.org/). The conserved motifs of expansin proteins were determined by the MEME online program (http://meme-suite.org/tools/meme), and the parameters were defined based on those used by Liu et al. [68]. The cis-acting elements that were 2000 bp upstream of all *FtEXPs* were predicted through the PlantCare online software (http://bioinformatics.psb.ugent.be/webtools/plantcare/html/).

### Chromosome distribution, duplication events, Syntenic analysis and Ka/Ks ratio calculations of EXPs to homologous gene pairs in all angiosperms

Gff files and sequencing files were used to obtain the chromosome localization information of *FtEXPs*. Analysis of *FtEXP* duplication events was performed through multiple collinear scanning toolkits (MCScanX) [69] and to visualize data through TBtools v1.082. The syntenic relationships between the expansin genes of Tartary buckwheat and five angiosperms were visualized by using Dual Synteny Plotter software and were visualized using TBtools v1.082. KaKs_Calculator 2.0 was used to calculate the synonymous (Ks) and nonsynonymous (Ka) substitutions of each homologous expansin gene pair and their ratios (Ka/Ks) [70].

### Plant growth

The big fruit Tartary buckwheat accessions (BTB, XIQIAO) and small fruit Tartary buckwheat accessions (STB, MIQIAO) were cultivated at the experimental farm of the College of Life Sciences, Sichuan Agricultural University, China. We collected samples from three replicate plants. After 90 days of Tartary buckwheat germination, we collected flowers, stems, roots and leaves. We picked the fruits at 13, 19 and 25 days after pollination (DAP). All picked materials were rapidly placed in liquid nitrogen and were then kept at − 80 °C.

### Auxin treatment of STB

In previous studies of the regulation of fruit size by *FtARF2*, STB was sprayed at the budding stage with 40, 70, 100, 130, or 160 mg L^− 1^ IAA. It was found that 100 mg L^− 1^ IAA was the best concentration for increasing fruit weight [68]. Therefore, STB plants were treated with 100 mg L^− 1^ IAA in this study. After treatment, fruits at 13 DAP and 19 DAP were collected and placed at − 80 °C.

### Expression analysis of the FtEXPs

The *FtEXP* expression in the stem, root, leaf, flower and fruit during different developmental stages (green fruit stage, 13 DAP; discoloration stage, 19 DAP; and initial maturity stage, 25 DAP) were measured by qRT-PCR. At the same time, the expression of *FtEXPA* genes in the BTB and STB fruits and in the STB fruits treated with IAA was also measured. The primers used in qRT-PCR (Table S8) were designed through the online software primer 3 (https://www.ncbi.nlm.nih.gov/tools/primer-blast/). The Tartary buckwheat histone H3 gene was used as an internal reference gene, and SYBR Premix Ex Taq II (TaKaRa) was used in qRT-PCR [71]. The correlative expression data were calculated using the 2^-△△CT^ method [72].

### Statistical analysis

The obtained experimental data were processed and analyzed by GraphPad Prism 7.04, and the least significant difference test was used to compare the data.

### Data availability statement

The datasets supporting the conclusions of this article are included in the article and its additional files.

## Supplementary Information

**Additional file 1: Figure S1.** Phylogenetic relationships and motif compositions of the expansin proteins from five different plant species. Outer layer: Phylogenetic trees were constructed using MEGA 7.0 with the maximum likelihood method. These phylogenetic trees were visualized by using the online tool Interactive Tree Of Life (iTOL) (http://itol2.embl.de/). Inner layer: Distribution of the conserved motifs in expansin proteins. The conserved motifs of the expansin proteins were determined by the MEME online program (http://meme-suite.org/tools/meme) and were visualized by TBtools v1.082. The differently colored boxes represent different motifs and their positions in each expansin protein sequence.

**Additional file 2: Figure S2.** Protein motif model of the expansin protein family in representative species. (A) Motif model of the algal expansin protein family. The conserved motifs of the algal expansin proteins were determined by the MEME online program (http://meme-suite.org/tools/meme) and were visualized by TBtools v1.082. (B) Motif model of the bryophyta expansin protein family. The conserved motifs of the bryophyta expansin proteins were determined by the MEME online program (http://meme-suite.org/tools/meme), and were visualized by TBtools v1.082. (C) Motif model of the monocotyledon expansin protein family. The conserved motifs of the monocotyledon expansin proteins were determined by the MEME online program (http://meme-suite.org/tools/meme), and were visualized by TBtools v1.082. (D) Motif model of the dicotyledonous expansin protein family. The conserved motifs of dicotyledonous expansin proteins were determined by the MEME online program (http://meme-suite.org/tools/meme), and were visualized by TBtools v1.082.

**Additional file 3: Figure S3.** Cis-acting element analysis of the expansin protein promoters from Tartary buckwheat. The cis-acting elements that were 2000 bp upstream of all *FtEXPs* were predicted through the PlantCare online software (http://bioinformatics.psb.ugent.be/webtools/plantcare/html/) and were visualized by TBtools v1.082. Blocks of different colors represent light responsiveness elements, low temperature responsiveness elements, salicylic acid responsiveness elements, abscisic acid responsiveness elements, MeJA responsiveness elements, auxin responsiveness elements, gibberellin responsiveness elements and defense and stress responsiveness elements.

**Additional file 4: Table S1.** Number of expansion family members in multiple species. **Table S2** Gene ID of expansion in multiple species. **Table S3** List of the 37 FtEXP genes identified in this study. **Table S4** List of the Tartary buckwheat 37 FtEXP genes identified in this study. **Table S5** Analysis and distribution of conserved motifs in Tartary buckwheat expansin proteins. **Table S6** Ka, Ks and Ka/Ks value of synteny expansin gene pairs in angiosperms genome. **Table S7** Synteny expansin gene pairs between Tartary buckwheat and other angiosperms. **Table S8** Primer sequences for qRT-PCR.

## Data Availability

The genome sequences of Tartary buckwheat used for identifying the FtEXPs in this study were located in the Tartary Buckwheat Genome Project (TBGP; http://www.mbkbase.org/Pinku1/). The Tartary buckwheat accessions (XIQIAO and MIQIAO) materials used in the experiment were supplied by Professor Wang Anhu of Xichang University. The datasets supporting the conclusions of this article are included with in the article and its Additional files.

## Abbreviations

*A. thaliana*: *Arabidopsis thaliana*
BTB: Big fruit Tartary buckwheat
DAP: Days after pollination
EXP: Expansin
EXPA: α-expansin
EXPB: β-expansin
EXLA: Expansin-like A
EXLB: Expansin-like B
FtEXP: *Fagopyrum tataricum* expansin
FtEXPA: FtEXP in EXPA family
Gff: Generic feature format
GH45: Glycoside hydro-lase family-45
HMM: Hidden Markov Model
IAA: Indole-3-acetic acid
Mw: Molecular weight
PI: Isoelectric points
qRT-PCR: Quantitative real-time polymerase chain reaction
STB: Small fruit Tartary buckwheat
TBGP: Tartary buckwheat genome project
