# Supplementary figures and images for: Evolutionary research on the expansin protein family during the plant transition to land provides new insights into the development of Tartary buckwheat fruit
Source: BMC Genomics. 2021 Apr 9;22:252. doi: 10.1186/s12864-021-07562-w (PMC8034093; doi:10.1186/s12864-021-07562-w)

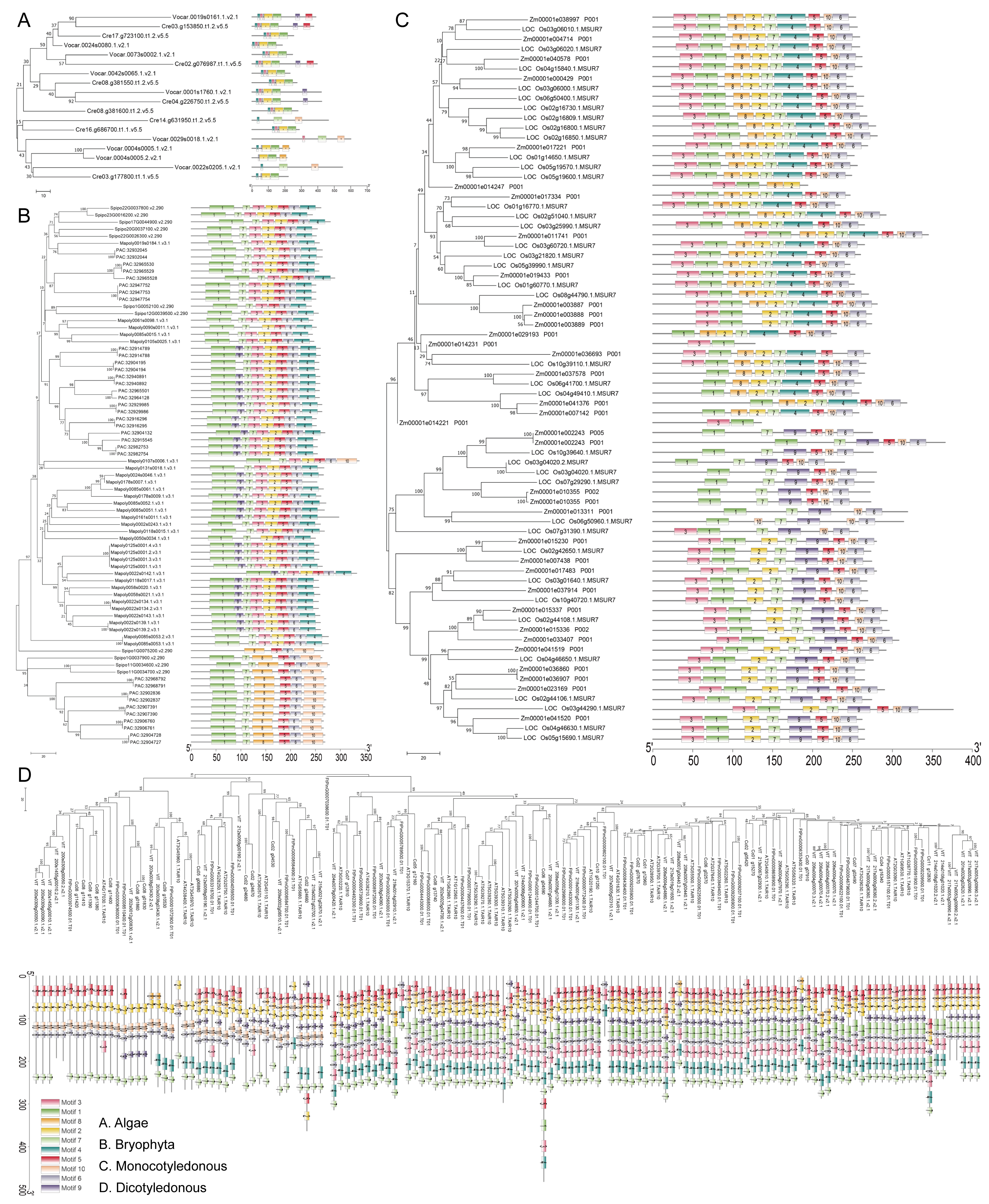

Supplement: Supplementary file 1 — Additional file 1: Figure S1. Phylogenetic relationships and motif compositions of the expansin proteins from five different plant species. Outer layer: Phylogenetic trees were constructed using MEGA 7.0 with the maximum likelihood method. These phylogenetic trees were visualized by using the online tool Interactive Tree Of Life (iTOL) (http://itol2.embl.de/). Inner layer: Distribution of the conserved motifs in expansin proteins. The conserved motifs of the expansin proteins were determined by the MEME online program (http://meme-suite.org/tools/meme) and were visualized by TBtools v1.082. The differently colored boxes represent different motifs and their positions in each expansin protein sequence. [file 12864_2021_7562_MOESM1_ESM.jpg]

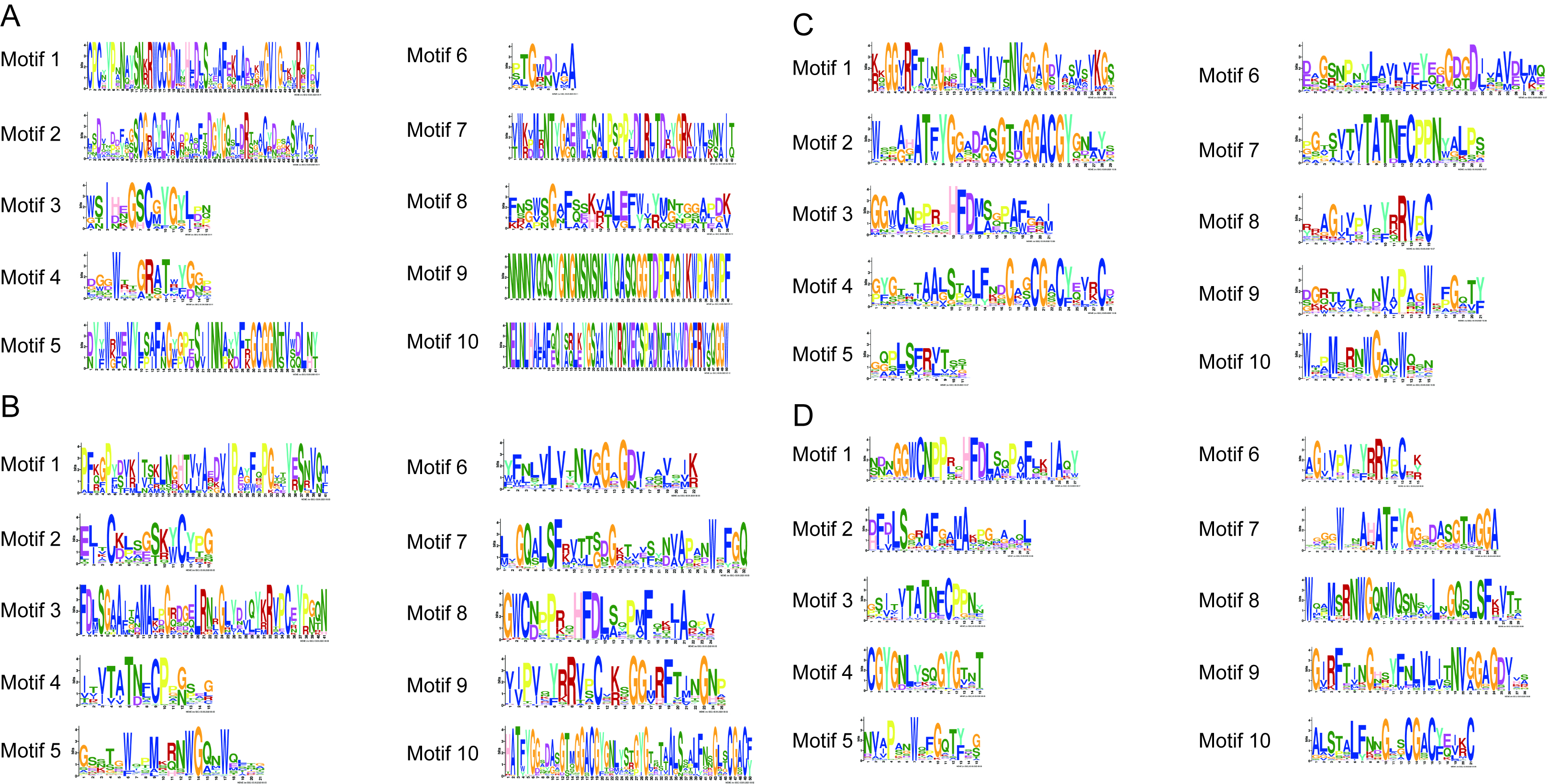

Supplement: Supplementary file 2 — Additional file 2: Figure S2. Protein motif model of the expansin protein family in representative species. (A) Motif model of the algal expansin protein family. The conserved motifs of the algal expansin proteins were determined by the MEME online program (http://meme-suite.org/tools/meme) and were visualized by TBtools v1.082. (B) Motif model of the bryophyta expansin protein family. The conserved motifs of the bryophyta expansin proteins were determined by the MEME online program (http://meme-suite.org/tools/meme), and were visualized by TBtools v1.082. (C) Motif model of the monocotyledon expansin protein family. The conserved motifs of the monocotyledon expansin proteins were determined by the MEME online program (http://meme-suite.org/tools/meme), and were visualized by TBtools v1.082. (D) Motif model of the dicotyledonous expansin protein family. The conserved motifs of dicotyledonous expansin proteins were determined by the MEME online program (http://meme-suite.org/tools/meme), and were visualized by TBtools v1.082. [file 12864_2021_7562_MOESM2_ESM.jpg]

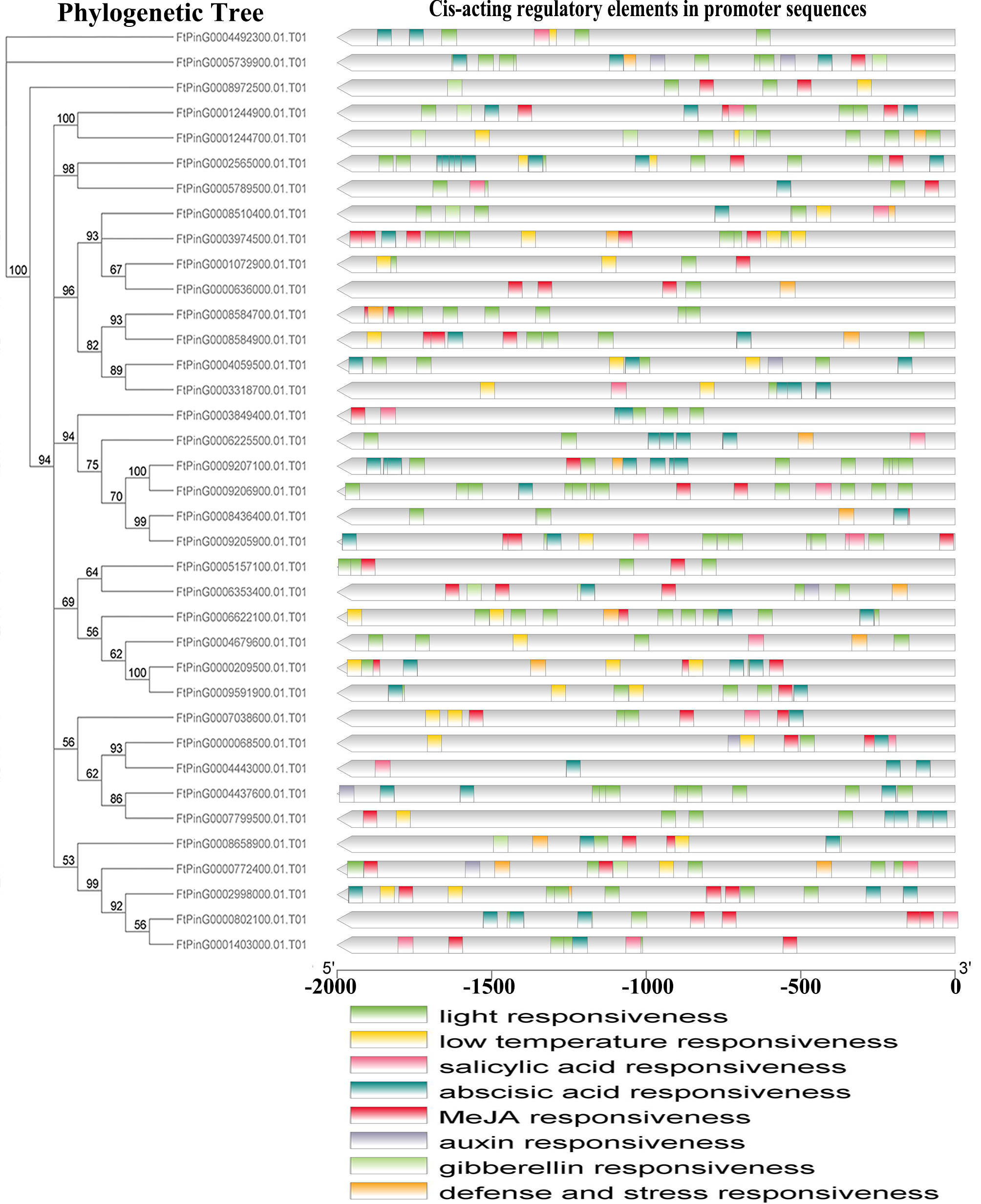

Supplement: Supplementary file 3 — Additional file 3: Figure S3. Cis-acting element analysis of the expansin protein promoters from Tartary buckwheat. The cis-acting elements that were 2000 bp upstream of all FtEXPs were predicted through the PlantCare online software (http://bioinformatics.psb.ugent.be/webtools/plantcare/html/) and were visualized by TBtools v1.082. Blocks of different colors represent light responsiveness elements, low temperature responsiveness elements, salicylic acid responsiveness elements, abscisic acid responsiveness elements, MeJA responsiveness elements, auxin responsiveness elements, gibberellin responsiveness elements and defense and stress responsiveness elements. [file 12864_2021_7562_MOESM3_ESM.jpg]
